# Retraction: Iso-pencillixanthone A from a marine-derived fungus reverses multidrug resistance in cervical cancer cells through down-regulating P-gp and re-activating apoptosis

**DOI:** 10.1039/d5ra90002f

**Published:** 2025-01-09

**Authors:** Li Chen, Xinxin Li, Miaomiao Cheng, Siyuan Wang, Qiuhong Zheng, Qinying Liu

**Affiliations:** a Institute of Biomedical and Pharmaceutical Technology, Fuzhou University Fuzhou 350002 P. R. China; b Fujian Provincial Key Laboratory of Tumor Biotherapy, Fujian Cancer Hospital, Fujian Medical University Cancer Hospital Fuzhou 350014 P. R. China zqh2858@foxmail.com liuqy@fjmu.edu.cn +86-591-8366-0063

## Abstract

Retraction of ‘Iso-pencillixanthone A from a marine-derived fungus reverses multidrug resistance in cervical cancer cells through down-regulating P-gp and re-activating apoptosis’ by Li Chen *et al.*, *RSC Adv.*, 2018, **8**, 41192–41206, https://doi.org/10.1039/C8RA09506J.

The Royal Society of Chemistry hereby wholly retracts this *RSC Advances* article due to concerns with the reliability of the data.

In the FACS data in Fig. 4c, the HeLa/VCR iso-PXA 1 (μM) panel and the HeLa VCR 1 (μM) panel are identical. The authors have repeated this experiment, but an independent expert has concluded that the sample quality is low, with a high level of debris and the gating appears to be incorrect and cannot be used as a replacement.

Given the significance of these concerns, the findings presented in this paper are no longer reliable.

The authors were informed about the retraction of the article but have not responded.

Signed: Laura Fisher, Executive Editor, *RSC Advances*

Date: 19th December 2024

